# Does Future Diabetes Risk Impair Current Quality of Life? A Cross-Sectional Study of Health-Related Quality of Life in Relation to the Finnish Diabetes Risk Score (FINDRISC)

**DOI:** 10.1371/journal.pone.0147898

**Published:** 2016-02-03

**Authors:** Saku Väätäinen, Henna Cederberg, Risto Roine, Sirkka Keinänen-Kiukaanniemi, Jouko Saramies, Hannu Uusitalo, Jaakko Tuomilehto, Janne Martikainen

**Affiliations:** 1 Pharmacoeconomics and Outcomes Research Unit (PHORU), School of Pharmacy, University of Eastern Finland, Kuopio, Finland; 2 Department of Medicine, Kuopio University Hospital, Kuopio, Finland; 3 Research Centre for Comparative Effectiveness and Patient Safety (RECEPS), Department of Health and Social Management, University of Eastern Finland, Kuopio, Finland; 4 Group Administration, University of Helsinki and Helsinki University Hospital, Helsinki, Finland; 5 Center for Life Course Health research, University of Oulu, Oulu, Finland; 6 Unit of Primary Health Care and Medical Research Center, Oulu University Hospital, Oulu, Finland; 7 South Karelia Central Hospital, Lappeenranta, Finland; 8 Department of Ophthalmology, SILK, School of Medicine, University of Tampere and TAUH Eye Center, Tampere Finland; 9 Centre for Vascular Prevention, Danube-University Krems, Austria; 10 Department of Chronic Disease Prevention, National Institute for Health and Welfare, Helsinki, Finland; 11 Diabetes Research Group, King Abdulaziz University, Jeddah, Saudi Arabia; 12 Dasman Diabetes Institute, Dasman, Kuwait; University of Leipzig, GERMANY

## Abstract

**Objectives:**

Present study examines the relationship between the estimated risk of developing type 2 diabetes (T2D) and health-related quality of life (HRQoL). We quantify the association between Finnish Diabetes Risk Score (FINDRISC) and HRQoL, and examine the potential use of FINDRISC as tool to evaluate HRQoL indirectly.

**Methods:**

We conducted a cross-sectional study comprising 707 Finnish people without a diagnosis of T2D between the ages of 51 and 75 years. The risk of developing T2D was assessed using the validated and widely used FINDRISC (range 0–26 points), and quality of life was measured using two preference-based HRQoL instruments (15D and SF-6D) and one health profile instrument (SF-36). Effects of the individual FINDRISC items and demographic and clinical characteristics, such as co-morbidities, on HRQoL were studied using multivariable Tobit regression models.

**Results:**

Low HRQoL was significantly and directly associated with the estimated risk of developing T2D. An approximate 4–5 point change in FINDRISC score was observed to be associated with clinically noticeable changes in the preference-based instrument HRQoL index scores. The association between HRQoL and the risk of developing T2D was also observed for most dimensions of HRQoL in all applied HRQoL instruments. Overall, old age, lack of physical activity, obesity, and history of high blood glucose were the FINDRISC factors most prominently associated with lower HRQoL.

**Conclusions:**

The findings may help the health care professionals to substantiate the possible improvement in glucose metabolism and HRQoL potentially achieved by lifestyle changes, and better convince people at high risk of T2D to take action towards healthier lifestyle habits. FINDRISC may also provide an accurate proxy for HRQoL, and thus by estimating the risk of T2D with the FINDRISC, information about patients’ HRQoL may also be obtained indirectly, when it is not feasible to use HRQoL instruments.

## Introduction

Health-related quality of life (HRQoL) is an evolving, multidimensional construct of physical, psychological and functional well-being that is increasingly used as an outcome in effectiveness research examining the effects of a disease or intervention on individuals’ health [1]. Particularly useful are preference-based HRQoL instruments (i.e., so-called health utility measures) that can be used to estimate quality-adjusted life years (QALYs) for cost-utility evaluations [2]. In essence, these measures are generic in the sense that they can be reliably applied to any disease entity and also consider population preferences concerning various health states.

Unhealthy lifestyle habits, such as poor diet and physical inactivity, are among the leading causes of mortality and disability in the Western world [3] and are also partly responsible for the increasing epidemic of type 2 diabetes (T2D) and other obesity-related morbidities. Although the preference-based HRQoL effects of T2D and prediabetes have been examined previously [4,5], the association between estimated diabetes risk and HRQoL is less understood. A few previous studies have reported an association between overall lifestyle and HRQoL [6,7], but no study has evaluated the association between the estimated T2D risk and HRQoL. While the effects of some individual lifestyle factors causing diabetes, such as physical inactivity [8] and obesity [9], on HRQoL have been examined before, it is clinically more relevant to examine the combined effect of multiple concurrent risk factors in multifactorial diseases such as T2D.

Several non-invasive screening questionnaires for assessing the risk of T2D have been developed in the past ten years [10,11]. Compared to invasive tools, these questionnaires provide a feasible method to routinely screen the population to detect individuals with either undetected T2D, abnormal glucose metabolism or an elevated risk to develop T2D in the future. Although most T2D risk questionnaires share similar characteristics and constructs, the Finnish Diabetes Risk Score (FINDRISC) [12] is currently one of the most widely validated and utilized T2D risk score [10,11].

Although the FINDRISC was originally developed to assess future T2D risk, subsequent studies have shown that it can also be used to detect prevalent abnormal glucose metabolism [13,14] and predict other significant health outcomes, such as coronary heart disease, stroke and overall mortality [15,16]. These conditions can have a profound impact on an individual’s expected life span and future quality of life, but a direct comparison of the FINDRISC and HRQoL scores has not been reported previously. Since FINDRISC is a feasible tool for estimating patients’ T2D risk in routine clinical practice, it could potentially also provide a simple way to evaluate patients HRQoL in clinical work and research, when the use of additional separate HRQoL questionnaires would not be feasible.

The present study had two aims. First, we sought to quantify how HRQoL may be associated with the estimated T2D risk by examining the relationship between the FINDRISC score and two validated preference-based HRQoL instruments, the 15D and the SF-6D, as well as one health profile instrument, SF-36. Second, we wanted to provide a method to estimate the HRQoL scores of these two preference-based instruments by using the FINDRISC score as a proxy.

## Methods

### Study Design, Study Population and Data Collection

**Fig 1:**
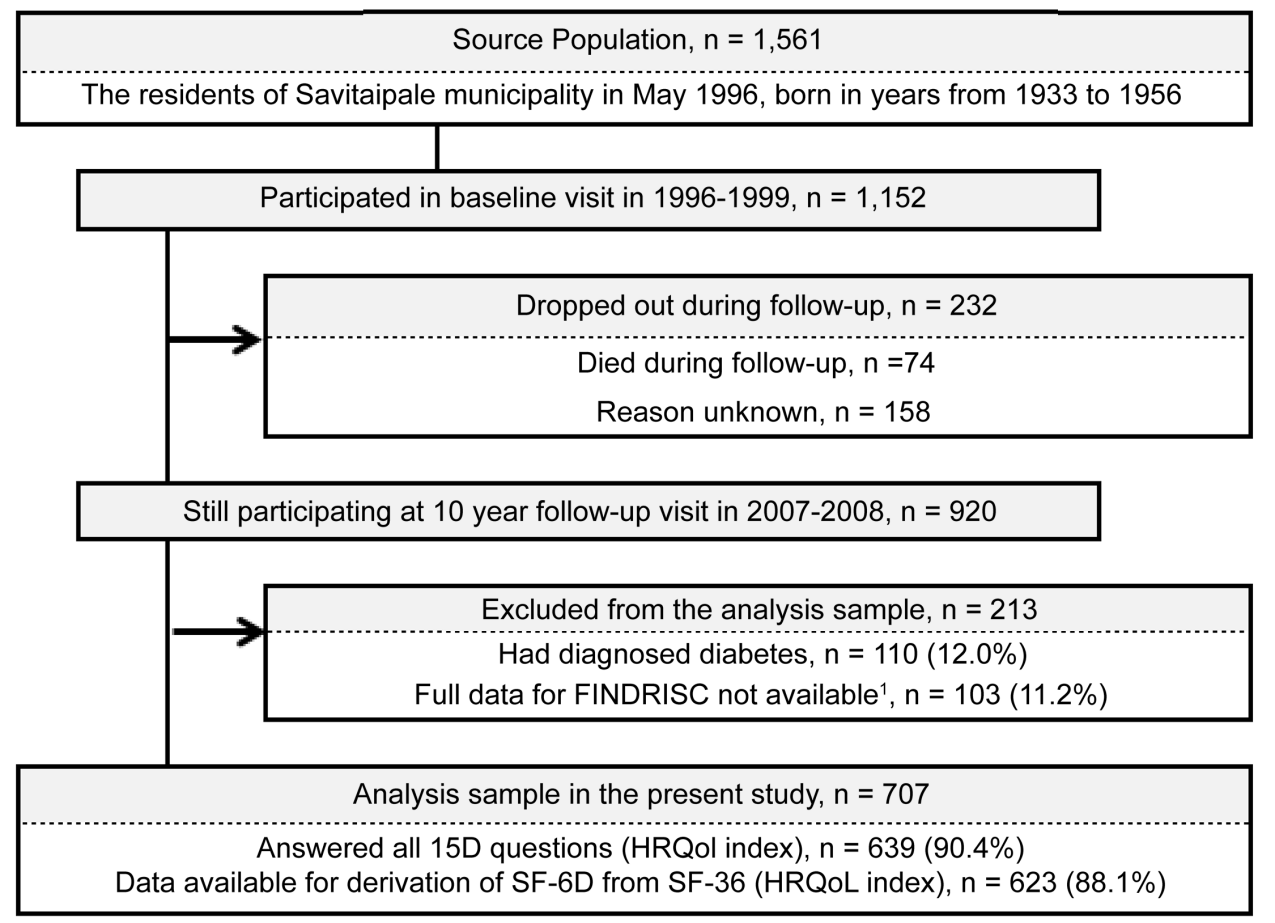
Flow diagram of the participants included in the study sample. FINDRISC: Finnish Diabetes Risk Score. 1) FINDRISC question regarding the consumption of fruits, berries and vegetables was omitted.

The analysis sample of the present study comprised 707 individuals who participated at the 10-year follow-up visit of the Savitaipale Study in 2007–2008 (Fig 1) [17]. The original study population in the longitudinal observational Savitaipale Study consisted of the residents of the municipality of Savitaipale, Finland who were born between 1933 and 1956. At the time of the 10-year follow-up visit, 74 individuals had died and 158 discontinued follow-up for unreported reasons. An additional 213 people were excluded from the analyses either because they had been diagnosed with diabetes (n = 110) or because full data to calculate the FINDRISC score were not available (n = 103).

The data were obtained through a questionnaire and laboratory assays and diagnostic tests during the study visit. All participants not treated with glucose-lowering drugs underwent an oral glucose tolerance test (OGTT), which was conducted and interpreted using the WHO 1999 classification criteria using plasma glucose. Additionally, a retrospective review of patient records was conducted for all participants, and co-morbidities were recorded using the Elixhauser co-morbidity index [18]. Depressive symptoms were evaluated with a Finnish translation of the original Beck Depression Inventory (BDI) [19]. Consequently, diabetes and depression were omitted from Elixhauser morbidities, as these were assessed separately.

All study participants gave their informed consent in writing prior to their inclusion in the study. All study protocols and informed consent procedures were approved by the ethics committee of the South Karelia District of Social and Health Services (decision number 24/98).

### Diabetes Risk

We examined the T2D risk using the FINDRISC, a validated and widely used composite T2D risk score questionnaire that comprises questions on age; body mass index (BMI); waist circumference; physical activity; consumption of fruits, berries or vegetables; and history of antihypertensive medication, history of high blood glucose and family history of diabetes [12]. The categorical response options are given weights (higher levels indicate greater significance) and then summed to obtain the total risk score. The total score ranges from 0 to 26, in which a higher score corresponds to a greater diabetes risk.

In the present study, the FINDRISC score was computed for each participant retrospectively. The item concerning the consumption of fruits, berries or vegetables was not included in the study questionnaire in a meaningful way and hence was omitted from the risk score. Although study participants underwent an OGTT, the history of a high blood glucose level was based on the participants’ own report, as intended in the original FINDRISC form.

### Health-Related Quality of Life

During the study visit, HRQoL was evaluated with two different questionnaires: the Finnish 15D [20] and the RAND-36 Item Health Survey v. 1.0 [21] (SF-36), which was also used to derive a preference-based index score SF-6D [22,23].

The 15D is a generic, standardized and self-administered preference-based measure of HRQoL and yields a single index score (also known as health utility), as well as a 15-dimensional HRQoL profile. The 15D comprises 15 questions (dimensions): mobility, vision, hearing, breathing, sleeping, eating, speech, excretion, usual activities, mental function, discomfort and symptoms, depression, distress, vitality and sexual activity; each has five answer options [20]. The index score is obtained by weighting these dimensional scores with population-based preference weights based on an application of the multi-attribute utility theory [20]. The 15D index and profile scores have values on a scale between 0 (representing HRQoL equal to being dead) and 1 (representing best possible HRQoL).

Although the 15D is a validated instrument and has been demonstrated to perform at least equally well as similar types of generic HRQoL instruments [24], we also employed the widely used SF-36 for wider comparability. The SF-36 is a 36-item questionnaire that is summarized in eight dimension scores: general health, bodily pain, emotional role limitation, physical role limitation, mental health, vitality, physical functioning and social functioning [21]. Each dimension score has values between 0 and 100, in which 0 means dead and 100 perfect health. Furthermore, the SF-36 was also used to derive its preference-based derivate, the SF-6D, as described by Brazier et al. [22,23]. As with the 15D, SF-6D values vary between 0 and 1.

To aid the practical interpretation of abstract HRQoL measures, the estimated minimum important differences (MID) can be used. Generally, the MID describes the minimum practically or clinically meaningful change in HRQoL score that an individual or a health care professional can notice. Alanne et al. [25] recently concluded that a difference of 0.015 or more can be considered the MID for the 15D index score. This value also corresponds well with an estimate based on 0.2xSD (standard deviation) as proposed by Fayers & Hays [26], if applied to our study sample (0.2*0.077 = 0.0154). The MID threshold of ≥ 0.027 for the SF-6D proposed by Luo et al. [27] concurs well with the above-mentioned 0.2xSD method applied to our study sample (0.2*0.115 = 0.0230) and thus seems comparable with the 15D’s MID estimate.

### Statistical Methods

Bivariate associations were examined using the Chi-squared test (categorical variables) and Jonckheere’s trend-test (ordinal vs. continuous variables). The relationship between the FINDRISC score and HRQoL was described graphically using means and confidence intervals (CI). The significance of linear trend between HRQoL and the FINDRISC score was assessed by fitting linear regression lines to the data.

To estimate the effects of the individual FINDRISC items, multivariable regression models were created using HRQoL index scores (15D and SF-6D, in separate models) as dependent variables and the FINDRISC items as independent variables. Tobit regression was used to account for the ceiling effect commonly associated with HRQoL measures [28]. For further analysis, age, sex, socioeconomic factors (cohabiting and employment status), and morbidities (depressive symptoms, glucose metabolism status, number of other morbidities) were included in the model. The variance in regression coefficients was estimated using the bootstrap procedure with 1,000 replications.

To scope the accuracy of model estimates, two metrics are reported. The percentage mean bias (and 95% confidence interval) in model predictions (ŷ) for each model is given by (ŷ –y) / y *100, where ŷ is the modeled estimate and y is the observed HRQoL score. In essence, this metric describes how much the model estimates differ from the actual observed values on a percentage scale. Additionally, we report squared root mean error (RMSE), $\sqrt{n^{-1}\;*\;\sum {\left(\hat{\text{y}}-\text{y}\right)}^{2}}$, which is a similar yet more widely utilized metric.

All analyses were conducted using Intercooled STATA 9.2, whereas IBM SPSS Statistics v. 21.0 and SAS 9.2 were also used to manage the data. The conventional value of *p* < 0.05 was used as the threshold for statistical significance.

## Results

### Descriptive Statistics

On average (SD), study subjects were 62.3 (6.7) years old, had average BMI of 26.4 (4.0) kg/m^2^. Overall, 44.0% of the study subjects were male. Although 24.5% of the subjects had at least two morbidities, 41.5% of the subjects did not have any morbidities identified by the Elixhauser index. The study sample characteristics are described in relation to FINDRISC categories in Table 1. The mean (SD) and median (range) FINDRISC scores were 14.8 (4.23) and 15 (2 to 25), respectively. Overall, 95% (n = 672/707) of the observed FINDRISC scores were between 6 and 22. The mean (SD) and median (range) 15D index values were 0.910 (0.077) and 0.926 (0.564 to 1), respectively. The mean and median SF-6D values were 0.770 (0.115) and 0.780 (0.487 to 1), respectively. The observed ceiling effect was modest in both HRQoL index measures: 8.5% (n = 54/639) and 3.2% (n = 20/623) of the study subjects had a HRQoL score of 1 in the 15D and SF-6D, respectively.

**Table 1:** The characteristics of the participants across the diabetes risk (FINDRISC) categories.

	Finnish Diabetes Risk Score (FINDRISC)										
	Less than 7		7–11		12–14		15–20		More than 20		
Variable	(n = 19)		(n = 165)		(n = 137)		(n = 324)		(n = 62)		p*
**Age**											
45 to 54 years	3	(15.8)	35	(21.2)	29	(21.2)	18	(5.6)	2	(3.2)	*< 0*.*001*
55 to 64 years	10	(52.6)	84	(50.9)	74	(54.0)	167	(51.5)	18	(29.0)	
Older than 64 years	6	(31.6)	46	(27.9)	34	(24.8)	139	(42.9)	42	(67.7)	
**Body mass index**	0	(0.0)	0	(0.0)	0	(0.0)	0	(0.0)	0	(0.0)	
Less than 25 kg/m^2^	18	(94.7)	129	(78.2)	53	(38.7)	80	(24.7)	0	(0.0)	*< 0*.*001*
25–30 kg/m^2^	1	(5.3)	34	(20.6)	77	(56.2)	181	(55.9)	24	(38.7)	
More than 30 kg/m^2^	0	(0.0)	2	(1.2)	7	(5.1)	63	(19.4)	38	(61.3)	
**Waist circumference**											
Less than 94 cm for men / 80 cm for women	16	(84.2)	127	(77.0)	20	(14.6)	47	(14.5)	0	(0.0)	*< 0*.*001*
94–102 cm for men / 80–88 cm for women	3	(15.8)	22	(13.3)	83	(60.6)	104	(32.1)	5	(8.1)	
More than 102 cm for men / 88 cm for women	0	(0.0)	16	(9.7)	34	(24.8)	173	(53.4)	57	(91.9)	
**Less than 30 minutes of daily physical activity**	0	(0.0)	8	(4.8)	10	(7.3)	30	(9.3)	13	(21.0)	*0*.*002*
**History of blood pressure medication**	14	(73.7)	140	(84.8)	98	(71.5)	275	(84.9)	58	(93.5)	*0*.*001*
**History of high blood glucose**	0	(0.0)	126	(76.4)	122	(89.1)	303	(93.5)	62	(100.0)	*< 0*.*001*
**Family diabetes**											
No history of family diabetes	19	(100.0)	160	(97.0)	117	(85.4)	126	(38.9)	0	(0.0)	*< 0*.*001*
2nd degree relative	0	(0.0)	0	(0.0)	1	(0.7)	6	(1.9)	1	(1.6)	
1st degree relative	0	(0.0)	5	(3.0)	19	(13.9)	192	(59.3)	61	(98.4)	
**Male**	12	(63.2)	84	(50.9)	59	(43.1)	135	(41.7)	21	(33.9)	*0*.*052*
**Single, separated or widowed**†	4	(21.1)	48	(29.6)	37	(27.0)	82	(25.7)	15	(24.6)	*0*.*857*
**Employment status**											
Employed full-time or part-time	13	(68.4)	110	(69.2)	92	(67.6)	176	(56.2)	16	(26.2)	*< 0*.*001*
Retired	5	(26.3)	41	(25.8)	29	(21.3)	94	(30.0)	34	(55.7)	
Unemployed or on disability pension	1	(5.3)	8	(5.0)	15	(11.0)	43	(13.7)	11	(18.0)	
Data missing	0		6		1		11		1		
**Glucose metabolism status**											
Normal glucose metabolism	15	(78.9)	123	(74.5)	99	(72.3)	232	(71.6)	31	(50.0)	*0*.*016*
Increased fasting glucose	3	(15.8)	11	(6.7)	15	(10.9)	26	(8.0)	6	(9.7)	
Impaired glucose tolerance	1	(5.3)	21	(12.7)	19	(13.9)	48	(14.8)	20	(32.3)	
Screen detected type 2 diabetes	0	(0.0)	10	(6.1)	4	(2.9)	18	(5.6)	5	(8.1)	
**Beck's Depression Inventory (BDI),** mean (SD)	4.3	(4.3)	4.9	(4.6)	5.6	(6.1)	5.8	(5.4)	7.12	(5.93)	*< 0*.*001*
**Number of morbidities,** mean (SD)	0.4	(0.5)	0.6	(0.8)	0.8	(0.9)	1.0	(1.0)	0.9	(1.0)	*< 0*.*001*

Data are presented as n (%), unless otherwise indicated. SD: Standard deviation. FINDRISC: Finnish Diabetes Risk Score

*) Chi-squared test for categorical variables, Jonckheere’s trend test for Beck's Depression Inventory and number of morbidities.

†) In relation to married or cohabited individuals, data missing for 0+3+0+5+1 = 9 individuals.

### Relationship between Overall Diabetes Risk and HRQoL

**Fig 2:**
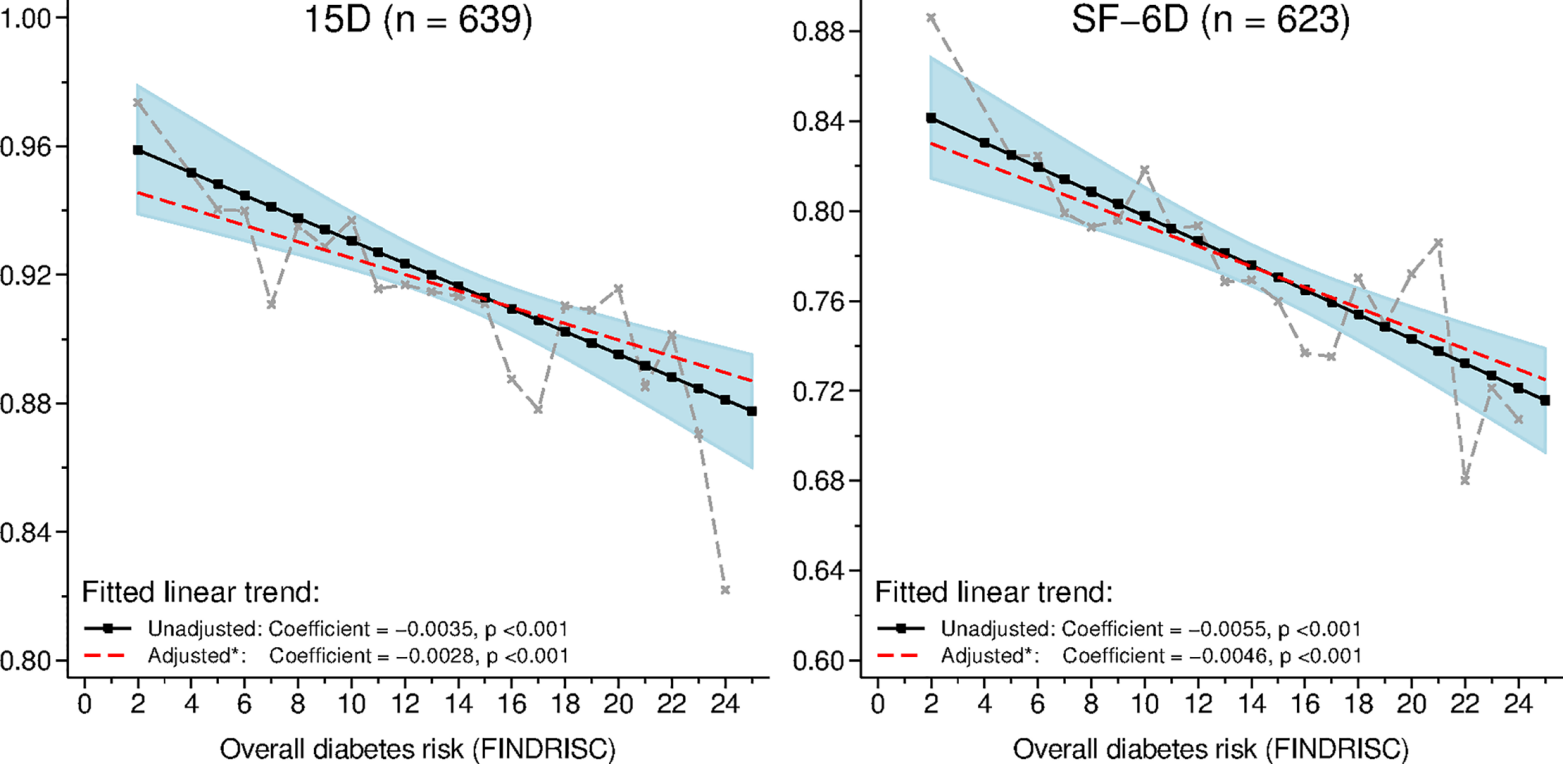
Observed mean HRQoL index values and fitted linear trends (95% CI) in relation to the FINDRISC scores. Linear trends were fitted to data using Tobit regression. The corresponding variance was estimated using a bootstrap procedure with 1,000 replications.*Adjusted for sex, employment and cohabiting. HRQoL: Health-related quality of life. FINDRISC: Finnish Diabetes Risk Score.

Both the 15D and SF-6D indices were significantly and directly associated with the FINDRISC score (Fig 2). The association was reasonably linear, and on average, a one-point increase in the FINDRISC score corresponded to a change of -0.0035 (95% CI: -0.0051 to -0.0020) and -0.0055 (-0.0076 to -0.0033) in the 15D and SF-6D scores, respectively. An approximate 4–5 point change in the FINDRISC score was associated with noticeable changes in both the 15D and SF-6D scores on average. Adjustment for sex, employment and cohabiting modified the associations slightly; the mean (95% CI) adjusted coefficients were -0.0028 (-0.0043 to -0.0014) and -0.0046 (-0.0068 to -0.0023) for the 15D and SF-6D, respectively.

**Fig 3:**
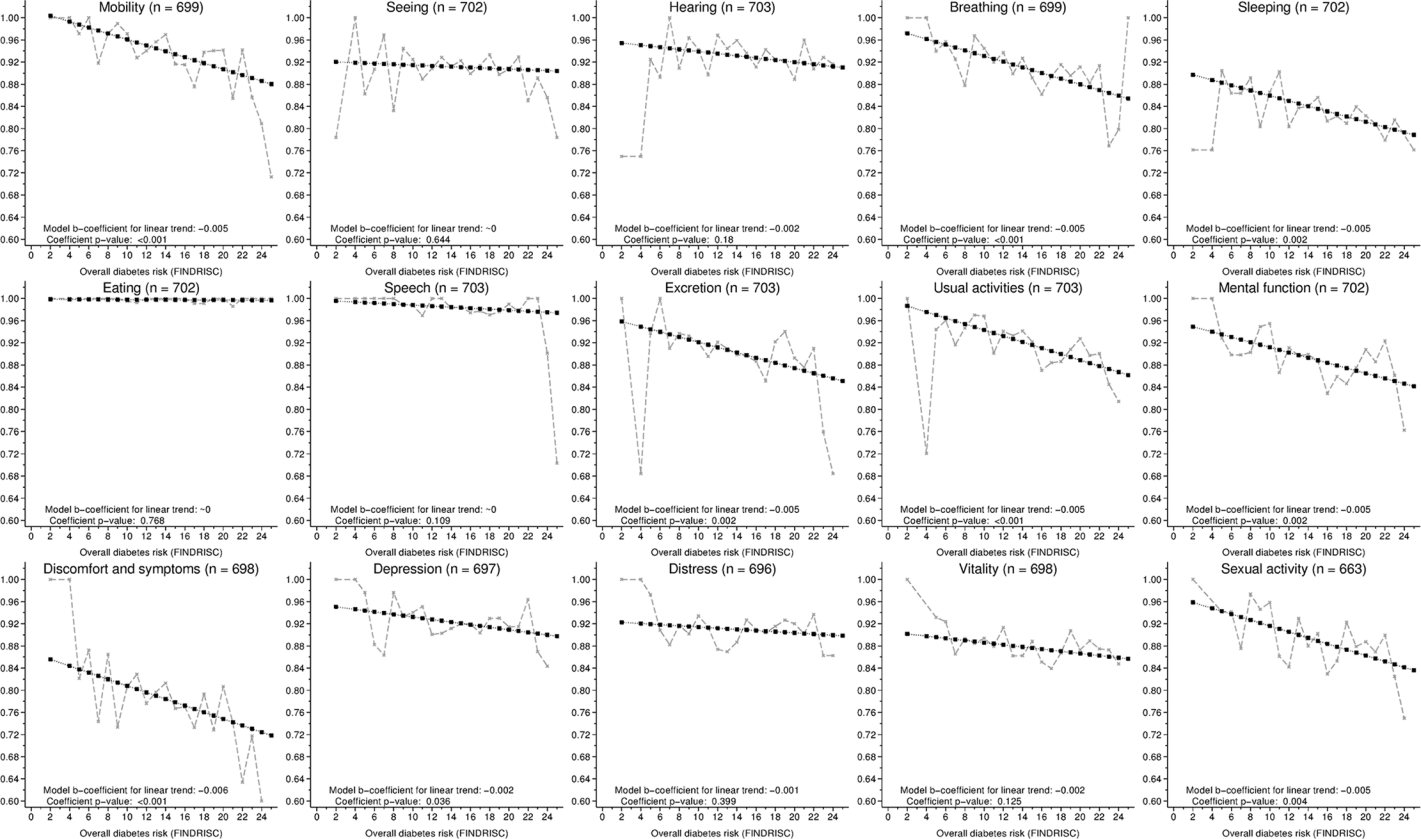
Mean observed HRQoL (15D) dimension values in relation to the FINDRISC score. Linear trends were fitted to data using ordinary least squares regression. The corresponding variance was estimated using a bootstrap procedure with 1,000 replications. HRQoL: Health-related quality of life. FINDRISC: Finnish Diabetes Risk Score.

**Fig 4:**
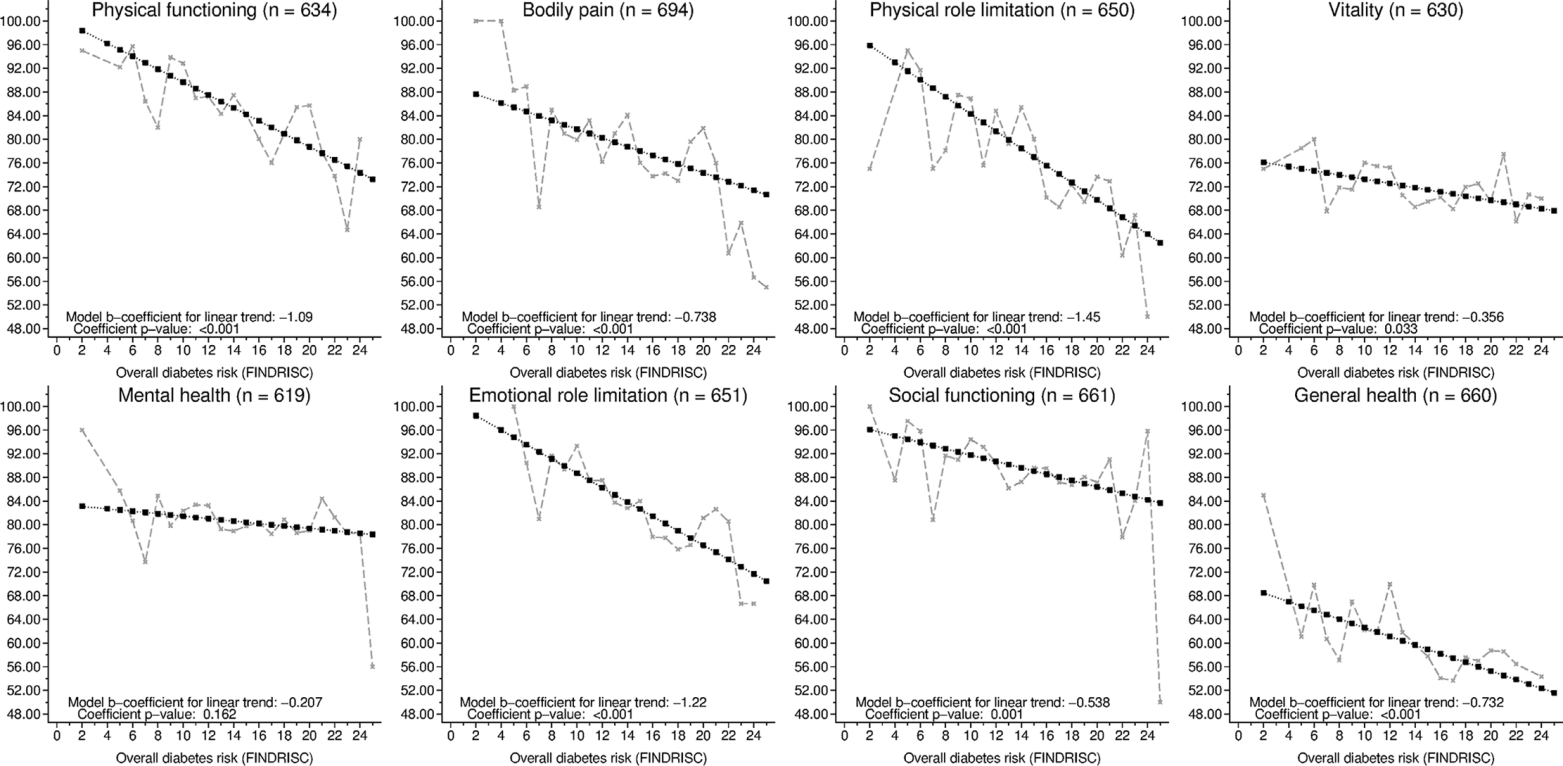
Mean observed HRQoL (SF-36) dimension values in relation to FINDRISC. Linear trends were fitted to data using ordinary least squares regression. The corresponding variance was estimated using a bootstrap procedure with 1,000 replications. HRQoL: Health-related quality of life. FINDRISC: Finnish Diabetes Risk Score.

An association between the HRQoL and FINDRISC score was observed for most dimensions of HRQoL. Whereas nine of fifteen 15D dimensions were significantly associated with the FINDRISC score, the sensory dimensions (seeing, hearing), ability to eat (eating) or speak (speech), and feelings of anxiety (distress) or vitality (vitality) were not (Fig 3). Of the SF-36 dimensions, all but mental health were significantly associated with the FINDRISC score (Fig 4). Although statistically significant, the association between the SF-36 vitality dimension and the FINDRISC score was noticeably weaker than that with other SF-36 dimensions.

### Individual Risk Factors and Their Impact on HRQoL

In the multivariable analysis, the findings were similar for the 15D (Table 2) and SF-6D (Table 3). Overall, old age, lack of physical activity, obesity and history of high blood glucose were the FINDRISC factors most prominently associated with lower HRQoL. When socioeconomic factors and morbidities were included in the model (full model), the associations between the FINDRISC items and HRQoL were weakened. A small bias (difference between model estimates and observed values) was present in all models, although it appeared to be negligible; the average bias ranged from 0.5% to 1.1% in the 15D models and 2.0% to 2.8% in the SF-6D.

**Table 2:** Marginal effects of FINDRISC items and other covariates on the 15D HRQoL index.

Variable	FINDRISC weight	FINDRISC items		Socioeconomic factors		Full model	
		b	(95% CI)	b	(95% CI)	b	(95% CI)
**Age**							
45 to 54 years	2	*ref*.		*ref*.		*ref*.	
55 to 64 years	3	-0.011	(-0.028; 0.005)	-0.005	(-0.021; 0.012)	0.002	(-0.011; 0.015)
Older than 64 years	4	-0.035***	(-0.053; -0.016)	-0.029**	(-0.051; -0.007)	-0.001	(-0.020; 0.017)
**Body mass index**							
Less than 25 kg/m^2^	0	*ref*.		*ref*.		*ref*.	
25–30 kg/m^2^	1	-0.004	(-0.021; 0.013)	-0.005	(-0.025; 0.014)	-0.007	(-0.020; 0.007)
More than 30 kg/m^2^	3	-0.029*	(-0.057; -0.001)	-0.029*	(-0.056; -0.001)	-0.017	(-0.038; 0.004)
**Waist circumference**							
Less than 94 cm for men / 80 cm for women	0	*ref*.		*ref*.		*ref*.	
94–102 cm for men / 80–88 cm for women	3	-0.001	(-0.019; 0.016)	0.003	(-0.015; 0.021)	0.000	(-0.013; 0.014)
More than 102 cm for men / 88 cm for women	4	-0.006	(-0.028; 0.016)	-0.003	(-0.026; 0.020)	0.002	(-0.015; 0.019)
**Less than 30 minutes of daily physical activity**	2	-0.048***	(-0.073; -0.022)	-0.042**	(-0.067; -0.017)	-0.023*	(-0.043; -0.003)
**History of blood pressure medication**	2	0.016	(-0.003; 0.035)	0.012	(-0.007; 0.031)	-0.006	(-0.020; 0.009)
**History of high blood glucose**	5	-0.018	(-0.035; 0.000)	-0.017	(-0.036; 0.002)	-0.015*	(-0.028; -0.003)
**Family diabetes**							
No history of family diabetes	0	*ref*.		*ref*.		*ref*.	
2^nd^ degree relative	3	-0.037	(-0.108; 0.033)	-0.043	(-0.110; 0.025)	-0.018	(-0.070; 0.035)
1^st^ degree relative	5	-0.006	(-0.019; 0.007)	-0.001	(-0.013; 0.012)	-0.005	(-0.015; 0.004)
**Male**				0.003	(-0.010; 0.016)	-0.011*	(-0.021; -0.001)
**Single, separated or widowed**†				0.003	(-0.012; 0.018)	0.003	(-0.009; 0.015)
**Employment status**							
Employed full-time or part-time				*ref*.		*ref*.	
Retired				-0.004	(-0.022; 0.014)	0.003	(-0.011; 0.016)
Unemployed or on disability pension				-0.072***	(-0.098; -0.046)	-0.037***	(-0.058; -0.017)
**Beck's Depression Inventory (BDI)**						-0.009***	(-0.010; -0.008)
**Number of morbidities**						-0.019***	(-0.026; -0.012)
**Glucose metabolism status**							
Normal glucose metabolism						*ref*.	
Increased fasting glucose						0.009	(-0.004; 0.023)
Impaired glucose tolerance						0.002	(-0.014; 0.018)
Screen detected type 2 diabetes						-0.014	(-0.041; 0.013)
**Constant**		0.949***	(0.922; 0.976)	0.949***	(0.921; 0.976)	1.010***	(0.988; 1.033)
**Number of observations**		639		619		603	
**BIC**		-1111.627		-1088.229		-1404.084	
**RMSE**		0.075		0.072		0.0537	
**Mean % bias (95% CI)**		1.1	(1.11; 1.16)	1.0	(0.98; 1.03)	0.5	(0.49; 0.52)

Tobit model bootstrapped standard errors (1,000 replications). HRQoL: Health-related quality of life. FINDRISC: Finnish Diabetes Risk Score. 95% CI: 95% Confidence intervals.BIC: Bayesian Information Criteria. RMSE: Root Mean Squared Error. Statistically significant at

*) p < 0.05

**) p < 0.01

***) p < 0.001.

†) In relation to married or cohabited individuals, data missing for 0+3+0+5+1 = 9 individuals

**Table 3:** Marginal effects of FINDRISC items and other covariates on the SF-6D HRQoL index.

Variable	FINDRISC weight	FINDRISC items			Socioeconomic factors			Full model	
		b	(95% CI)		b	(95% CI)		b	(95% CI)
**Age**									
45 to 54 years	2	*ref*.			*ref*.			*ref*.	
55 to 64 years	3	-0.014	(-0.041; 0.013)		-0.009	(-0.038; 0.021)		-0.001	(-0.025; 0.023)
Older than 64 years	4	-0.040**	(-0.069; -0.012)		-0.041*	(-0.076; -0.006)		-0.005	(-0.036; 0.027)
**Body mass index**									
Less than 25 kg/m2	0	*ref*.			*ref*.			*ref*.	
25–30 kg/m2	1	-0.003	(-0.031; 0.025)		-0.010	(-0.038; 0.018)		-0.012	(-0.036; 0.013)
More than 30 kg/m2	3	-0.029	(-0.069; 0.011)		-0.035	(-0.075; 0.006)		-0.017	(-0.052; 0.018)
**Waist circumference**									
Less than 94 cm for males / 80 cm for females	0	*ref*.			*ref*.			*ref*.	
94–102 cm for males / 80–88 cm for females	3	-0.020	(-0.048; 0.008)		-0.009	(-0.038; 0.019)		-0.012	(-0.037; 0.012)
More than 102 cm for males / 88 cm for females	4	-0.021	(-0.057; 0.015)		-0.008	(-0.046; 0.030)		-0.002	(-0.035; 0.031)
**Less than 30 minutes of daily exercise**	2	-0.053**	(-0.087; -0.019)		-0.047**	(-0.080; -0.013)		-0.025	(-0.052; 0.002)
**History of blood pressure medication**	2	0.022	(-0.002; 0.047)		0.018	(-0.005; 0.042)		-0.003	(-0.023; 0.017)
**History of high blood glucose**	5	-0.036**	(-0.060; -0.012)		-0.037**	(-0.064; -0.010)		-0.030**	(-0.053; -0.007)
**Family diabetes**		*ref*.			*ref*.			*ref*.	
No history of family diabetes	0								
2nd degree relative	3	-0.044	(-0.095; 0.007)		-0.055*	(-0.106; -0.004)		-0.029	(-0.074; 0.016)
1st degree relative	5	-0.008	(-0.026; 0.010)		-0.006	(-0.025; 0.013)		-0.013	(-0.029; 0.003)
**Male**					0.022*	(0.003; 0.041)		0.007	(-0.011; 0.026)
**Single, separated or widowed**†					-0.004	(-0.026; 0.018)		-0.002	(-0.020; 0.016)
**Employment status**									
Employed full-time or part-time					*ref*.			*ref*.	
Retired					0.012	(-0.016; 0.039)		0.019	(-0.005; 0.044)
Unemployed or on disability pension					-0.070***	(-0.098; -0.042)		-0.031*	(-0.057; -0.006)
**Beck's Depression Inventory (BDI)**								-0.011***	(-0.012; -0.009)
**Number of morbidities**								-0.019***	(-0.027; -0.010)
**Glucose metabolism status**									
Normal glucose metabolism								*ref*.	
Increased fasting glucose								-0.008	(-0.040; 0.023)
Impaired glucose tolerance								-0.002	(-0.028; 0.024)
Screen detected type 2 diabetes								-0.040*	(-0.079; -0.001)
**Constant**		0.834***	(0.794; 0.875)		0.826***	(0.783; 0.869)		0.896***	(0.859; 0.932)
**Number of observations**	** **	623			601			586	
**BIC**		-773.1708			-748.9918			-903.6617	
**RMSE**		0.1127			0.1111			0.0931	
**Mean % bias (95% CI)**	** **	2.8	(2.73; 2.82)		2.7	(2.66; 2.75)		2.0	(1.95; 2.03)

Tobit model, bootstrapped standard errors (1,000 replications). HRQoL: Health-related quality of life. FINDRISC: Finnish Diabetes Risk Score. 95% CI: 95% Confidence intervals. BIC: Bayesian Information Criteria. RMSE: Root Mean Squared Error Statistically significant at

* p<0.05

** p<0.01

*** p<0.001.

†) In relation to married or cohabited individuals, data missing for 0+3+0+5+1 = 9 individuals.

The results of the multivariable models presented here should be interpreted with caution, as they represent the independent associations (i.e. marginal effects) between each individual model covariate and HRQoL measures, when the average effects of all other covariates included in the model have been adjusted for. For instance, this means that less than 30 minutes of daily physical activity is independently associated with a mean (95% CI) decrease of 0.025 (-0.052 to 0.002) in HRQoL, regardless of the patient’s age, BMI, depressive symptoms, number of morbidities or any other variable included in the model. However, risk factors for T2D and morbidities associated with T2D are rarely independently present. Instead, multiple risk factors are inherently concurrently present as FINDRISC scores increase. Whereas the individual FINDRISC items are naturally more prevalent at higher FINDRISC scores, the prevalence of abnormal glucose metabolism, depressive symptoms and the number of other morbidities was also significantly increased with a higher FINDRISC score (Table 1). Additionally, higher BMI categories alone were significantly associated with a lack of physical activity (Pearson’s chi-squared test: Chi2 = 15.48, p < 0.001), prevalence of abnormal glucose metabolism (Chi2 = 10.55, p = 0.005), as well as higher degree of depressive symptoms (Jonckeere’s trend test: J* = 2.03, p = 0.021) and number of other morbidities (J* = 4.817, p <0.001).

## Discussion

This study is the first to examine the relationship between HRQoL and the estimated diabetes risk using the FINDRISC, a widely validated and utilized non-invasive diabetes risk assessment tool. We found that the FINDRISC score was inversely associated with HRQOL indices overall, as well as with most dimensions of both the 15D and SF-36. Our findings have two important practical implications. First, the FINDRISC has the potential to be a readily applicable and feasible proxy measure for HRQOL in both research and clinical purposes. This would allow a practical way to evaluating HRQOL, when it is not feasible to burden patients with additional HRQOL questionnaires, as well as allow additional post-hoc analyses when HRQOL questionnaires have not been employed. Second, our findings suggest that FINDRISC could potentially to be used to quantify the potential HRQoL -impact of the life style changes. Our data show that the 4 to 5 point decrease in FINDRISC is associated with clinically noticeable changes in the preference-based instrument HRQoL index scores. This could help health care professionals substantiate the potential benefits of life style change to their patients.

Our findings are supported by another study that reported an association between overall unhealthy lifestyle habits and poor 15D score [7]. Furthermore, our findings are also supported by previous studies that have showed associations between poor HRQoL and physical inactivity [8], obesity [9] and overall life-style pattern [6].

Our observation that BMI and waist circumference were not associated with poor HRQoL after the data were adjusted for depressive symptoms, number of morbidities and current glucose metabolism status suggest that obesity itself is associated with a higher degree of various co-morbidities that reduce quality of life. Furthermore, the results of our multivariable analyses also suggest that the poor HRQoL associated with prediabetic stages in previous studies [4,5] may be mediated through underlying physical inactivity and obesity. In addition, it should be also noted, that the physical inactivity and obesity are themselves induced by some underlying cause–be it a physical condition preventing adequate exercise or some psychological factor. Overall, in clinical practice, it is always important to identify the unique key factors contributing to patient’s individual health status in order to aid patient effectively.

Interestingly, our data show that a previously detected high blood glucose level is statistically and clinically significantly associated with lower HRQoL on both the 15D and SF-6D, regardless of the current glucose metabolism status. Whereas the OGTT is a valid tool for describing a patient’s current glucose metabolism status, a self-reported history of high blood glucose may indicate ongoing problems in glucose metabolism and may also reflect the individual’s worry about their own health and wellbeing.

The present study has strengths and some potential weaknesses. First, we applied widely used and validated instruments for both diabetes risk and HRQoL. Second, we examined HRQoL with three different instruments, covering both preference-based HRQoL as well as more descriptive HRQoL profiles. Additionally, we also considered the potential HRQoL effects of socioeconomic factors and co-morbidity. The greatest weakness of the present study was the cross-sectional setting, which did not allow us to examine the changes in HRQoL over time in people with various FINDRISC scores. This prevents us from making any conclusions on the causal pathways. A longitudinal examination of HRQoL in relation to the FINDRISC score may also tell more about its validity as a predictor of future HRQoL. Additionally, our study sample only comprised middle-aged and older individuals from one Finnish municipality. Although this can be seen as a weakness, the study sample is representative with a high participation rate and comprises a homogenous set of individuals from the age strata in which the prevalence of glucose metabolism disorders is highest.

The FINDRISC is a comprehensive yet non-invasive tool that can be used to estimate the risk of T2D and other disorders of glucose metabolism [12–14], as well as many other morbidities and total mortality [15,16]. Previous studies have shown that a lifestyle intervention can effectively prevent the onset of T2D [29] in patients with impaired glucose tolerance, especially among individuals with a high FINDRISC score [30]. The findings of the present study indicate that the FINDRISC is also inversely associated with the patients current HRQoL. On the other hand, previous studies have demonstrated that the self-perceived health status is associated with current [31] and future [32] impairments in glucose metabolism. By substantiating the possible improvement in the glucose metabolism status and HRQoL achieved by lifestyle changes, health care professionals may better convince people at high risk of T2D to take action towards healthier lifestyle habits.

Our findings suggest that the FINDRISC may provide an accurate proxy for HRQoL at least at the population level, although the accuracy and validity of the FINDRISC as a proxy for individual-level HRQoL should be further evaluated in the future studies.

## References

[1] Wilson IB, Cleary PD. Linking clinical variables with health-related quality of life. A conceptual model of patient outcomes. JAMA. 1995 1 4;273(1):59–65. 7996652

[2] Drummond MF, Sculpher MJ, Torrance GW, O’Brien BJ, Stoddart GL. Methods for the Economic Evaluation of Health Care Programmes. 3 edition Oxford; New York: Oxford University Press; 2005. 396 p.

[3] World Health Organization. Global health risk: mortality and burden of disease attributable to selected major risk. Geneva: WHO; 2009.

[4] Väätäinen S, Keinänen-Kiukaanniemi S, Saramies J, Uusitalo H, Tuomilehto J, Martikainen J. Quality of life along the diabetes continuum: a cross-sectional view of health-related quality of life and general health status in middle-aged and older Finns. Qual Life Res. 2014 2 8;23(7):1935–44. 10.1007/s11136-014-0638-3 24510623

[5] Neumann A, Schoffer O, Norström F, Norberg M, Klug SJ, Lindholm L. Health-related quality of life for pre-diabetic states and type 2 diabetes mellitus: a cross-sectional study in Västerbotten Sweden. Health Qual Life Outcomes. 2014 10 24;12(1):150. 25342083 10.1186/s12955-014-0150-z PMC4212131

[6] Sabia S, Singh-Manoux A, Hagger-Johnson G, Cambois E, Brunner EJ, Kivimaki M. Influence of individual and combined healthy behaviours on successful aging. CMAJ Can Med Assoc J J Assoc Medicale Can. 2012 12 11;184(18):1985–92. 10.1503/cmaj.121080 PMC3519184 23091184

[7] Savolainen J, Kautiainen H, Miettola J, Niskanen L, Mäntyselkä P. Low quality of life and depressive symptoms are connected with an unhealthy lifestyle. Scand J Public Health. 2014 3;42(2):163–70. 10.1177/1403494813504837 24048729

[8] Anokye NK, Trueman P, Green C, Pavey TG, Taylor RS. Physical activity and health related quality of life. BMC Public Health. 2012 8 7;12(1):624. 22871153 10.1186/1471-2458-12-624 PMC3490805

[9] Keating CL, Peeters A, Swinburn BA, Magliano DJ, Moodie ML. Utility-based quality of life associated with overweight and obesity: the Australian diabetes, obesity, and lifestyle study. Obes Silver Spring Md. 2013 3;21(3):652–5. 10.1002/oby.20290 23592675

[10] Schwarz PE, Li J, Lindstrom J, Tuomilehto J. Tools for predicting the risk of type 2 diabetes in daily practice. Horm Metab Res Horm Stoffwechselforschung Horm Métabolisme. 2009;41(2):86–97. 10.1055/s-0028-1087203 19021089

[11] Brown N, Critchley J, Bogowicz P, Mayige M, Unwin N. Risk scores based on self-reported or available clinical data to detect undiagnosed Type 2 Diabetes: A systematic review. Diabetes Res Clin Pract. 2012;98(3):369–85. 10.1016/j.diabres.2012.09.005 23010559

[12] Lindström J, Tuomilehto J. The diabetes risk score: A practical tool to predict type 2 diabetes risk. Diabetes Care. 2003;26(3):725–31. 12610029 10.2337/diacare.26.3.725

[13] Saaristo T, Peltonen M, Lindström J, Saarikoski L, Sundvall J, Eriksson JG, et al Cross-sectional evaluation of the Finnish Diabetes Risk Score: A tool to identify undetected type 2 diabetes, abnormal glucose tolerance and metabolic syndrome. Diab Vasc Dis Res. 2005;2(2):67–72. 16305061 10.3132/dvdr.2005.011

[14] Zhang L, Zhang Z, Zhang Y, Hu G, Chen L. Evaluation of Finnish Diabetes Risk Score in Screening Undiagnosed Diabetes and Prediabetes among U.S. Adults by Gender and Race: NHANES 1999–2010. PLoS ONE. 2014 toukokuu;9(5):e97865 10.1371/journal.pone.0097865 24852786 PMC4031122

[15] Silventoinen K, Pankow J, Lindström J, Jousilahti P, Hu G, Tuomilehto J. The validity of the Finnish Diabetes Risk Score for the prediction of the incidence of coronary heart disease and stroke, and total mortality. Eur J Cardiovasc Prev Rehabil. 2005;12(5):451–8. 16210931 10.1097/01.hjr.0000174793.31812.21

[16] Heidemann C, Boeing H, Pischon T, Nöthlings U, Joost H-G, Schulze MB. Association of a diabetes risk score with risk of myocardial infarction, stroke, specific types of cancer, and mortality: A prospective study in the European Prospective Investigation into Cancer and Nutrition (EPIC)-Potsdam cohort. Eur J Epidemiol. 2009;24(6):281–8. 10.1007/s10654-009-9338-7 19357973

[17] Saramies J. Risk factors of type 2 diabetes and screening of abnormal glucose metabolism in primary health care. PhD Thesis, University of Kuopio, Finland; 2005.

[18] Elixhauser A, Steiner C, Harris DR, Coffey RM. Comorbidity Measures for Use with Administrative Data. Med Care. 1998;36(1):8–27. 9431328 10.1097/00005650-199801000-00004

[19] Beck A., T., Ward C., H., Mendelson M. et al An inventory for measuring depression. Arch Gen Psychiatry. 1969;4:561–71. 10.1001/archpsyc.1961.01710120031004 13688369

[20] Sintonen H. The 15D instrument of health-related quality of life: Properties and applications. Ann Med. 2001;33(5):328–36. 11491191 10.3109/07853890109002086

[21] Hays RD, Sherbourne CD, Mazel RM. The RAND 36-Item Health Survey 1.0. Health Econ. 1993;2(3):217–27. 8275167 10.1002/hec.4730020305

[22] Brazier J, Usherwood T, Harper R, Thomas K. Deriving a preference-based single index from the UK SF-36 Health Survey. J Clin Epidemiol. 1998;51(11):1115–28. 9817129 10.1016/s0895-4356(98)00103-6

[23] Brazier J, Roberts J, Deverill M. The estimation of a preference-based measure of health from the SF-36. J Health Econ. 2002;21(2):271–92. 11939242 10.1016/s0167-6296(01)00130-8

[24] Hawthorne G, Richardson J, Day NA. A comparison of the Assessment of Quality of Life (AQoL) with four other generic utility instruments. Ann Med. 2001;33(5):358–70. 11491195 10.3109/07853890109002090

[25] Alanne S, Roine RP, Räsänen P, Vainiola T, Sintonen H. Estimating the minimum important change in the 15D scores. Qual Life Res. 2014 8 22;1–8. 10.1007/s11136-014-0787-4 25145637

[26] Fayers PM, Hays RD. Don’t middle your MIDs: regression to the mean shrinks estimates of minimally important differences. Qual Life Res. 2014 2 1;23(1):1–4. 10.1007/s11136-013-0443-4 23722635 PMC3961825

[27] Luo N, Johnson JA, Coons SJ. Using instrument-defined health state transitions to estimate minimally important differences for four preference-based health-related quality of life instruments. Med Care. 2010;48(4):365–71. 20355266 10.1097/mlr.0b013e3181c162a2

[28] Austin PC, Escobar M, Kopec JA. The use of the Tobit model for analyzing measures of health status. Qual Life Res. 2000;9(8):901–10. 11284209 10.1023/a:1008938326604

[29] Lindström J, Peltonen M, Eriksson JG, Ilanne-Parikka P, Aunola S, Keinänen-Kiukaanniemi S, et al Improved lifestyle and decreased diabetes risk over 13 years: long-term follow-up of the randomised Finnish Diabetes Prevention Study (DPS). Diabetologia. 2013 2 1;56(2):284–93. 10.1007/s00125-012-2752-5 23093136

[30] Lindström J, Peltonen M, Eriksson JG, Aunola S, Hämäläinen H, Ilanne-Parikka P, et al Determinants for the Effectiveness of Lifestyle Intervention in the Finnish Diabetes Prevention Study. Diabetes Care. 2008 5 1;31(5):857–62. 10.2337/dc07-2162 18252900

[31] Andersson S, Ekman I, Friberg F, Daka B, Lindblad U, Larsson CA. The association between self-rated health and impaired glucose tolerance in Swedish adults: A cross-sectional study. Scand J Prim Health Care. 2013 4 29;31(2):111–8. 10.3109/02813432.2013.784541 23621319 PMC3656394

[32] Tapp RJ, O’Neil A, Shaw JE, Zimmet PZ, Oldenburg BF. Is there a link between components of health-related functioning and incident impaired glucose metabolism and type 2 diabetes? The Australian diabetes obesity and lifestyle (AusDiab) study. Diabetes Care. 2010;33(4):757–62. 10.2337/dc09-1107 20007943 PMC2845023

